# Adsorption of Strontium onto Adaxial and Abaxial Cuticle of *Photinia serrulata* Leaf

**DOI:** 10.3390/ijerph17031061

**Published:** 2020-02-07

**Authors:** Yungui Li, Xiang Luo, Xueying Bai, Wenxuan Lv, Yang Liao

**Affiliations:** 1 Department of Environmental Engineering, Southwest University of Science and Technology, Mianyang 621010, China; lx_luoxiang@163.com (X.L.); 15036027220@163.com (X.B.); wenxuan2807940648@163.com (W.L.);; 2 Sichuan Provincial Sci-Tech Cooperation Base of Low-cost Wastewater Treatment Technology, Southwest University of Science and Technology, Mianyang 621010, China

**Keywords:** leaf cuticle, strontium, adsorption, adaxial, abaxial

## Abstract

Leaf cuticle sorption is one important process for the uptake of environment pollutants in plants, and mixed powder including adaxial and abaxial cuticle is generally used to demonstrate the sorption behavior. However, the difference of adaxial and abaxial cuticle on plant cuticle sorption is not well understood. Abaxial cuticle (PAC) and adaxial cuticle (PBC) were isolated from hypostomatic *Photinia serrulata* to investigate their adsorption of a model radionuclide (strontium). The elemental composition and FTIR spectra for two cuticles were quite similar and both show high affinity (H/C, 1.59 and 1.65) and polarity ((O + N)/C, 0.470 and 0.499). Both adsorption isotherms fit well with Langmuir model (*R*^2^, 0.97 and 0.97), and the maximum adsorption capacity of PAC was 12.1 mg/g, little higher than that of PBC (10.3 mg/g). Adsorption of strontium increased with the increase of pH, and the maximum was attained when pH ≥4. Electrostatic attraction was demonstrated to be the main mechanism of -strontium adsorption onto PAC and PBC, and the similar adsorption of adaxial and abaxial cuticle was consistent with their similar isoelectric point.

## 1. Introduction

With the rapid development of global industrialization, especially the exacerbation of the energy crisis, nuclear energy has undergone dramatic progress. Up to now, more than 400 nuclear power plants have been put into operation, and the consequent threats to the environment have raised widespread concerns, as the three major nuclear disasters occurred in Three Mile Island, Chernobyl, and Fukushima brought about severe damage to human health and ecological safety [1,2,3]. Plants adsorption, primarily via root and foliar uptakes, is not only the beginning of the transmission of nuclides in the food chain, but also an important route of human and animal exposure to radionuclides [4,5,6]. Madoz-Escande et al. [7] found that, within one year after nuclear leak, the nuclides in the bodies of plants were mostly absorbed via foliar uptake rather root uptake. For some radionuclides (e.g., ^131^I and ^125^I), the non-root uptake is a primary and even the only channel to enter the plants. One month after the nuclear meltdown in Fukushima, the absorbed radiation level in plants 35 km away was 10 times higher than that in the soil. As a primary means of long-range migration of pollutants into the plants, foliar uptake is also crucial to the “sink” mechanism of pollutants and the safety of agricultural products [8,9,10].

Leaf cuticle, the film-like structure composed of wax, cutin, cutan and polysaccharide, is the first barrier that contacts with and absorbs nuclides, as well as a vital carrier of environmental pollutants accumulation [11,12,13]. Through investigation on the nuclear leak at Fukushima Plant, Nishikiori et al. [10] found that 60%–80% of ^137^Cs on the leaves of cedar were adhered on the epidermis, while 20%–40% existed inside the leaves and could be transferred from the old leaves to new ones. Cuticle adsorption is a fundamental process of foliar uptake and accumulation, and the adsorption rate and capacity will greatly impact on the uptake and accumulation of contaminants. So far, the existing research on cuticle adsorption have mostly focused on organic contaminants [11,14,15], while the cuticle adsorption of nuclides is rarely reported, and its mechanism remains unclear.

At present, studies on cuticle adsorption mainly investigated the mixed cuticle, i.e., with the adaxial and abaxial foliar cuticles unseparated [11,12,13]. However, there remain some differences in structure between the adaxial and abaxial foliar cuticles of most plants, especially angiosperms, the stomas of which are typically located in the abaxial epidermis [9]. The adaxial and abaxial cuticular topographies are distinct from each other resulting in different pollutant uptake behaviors [16,17]. Stomata located in abaxial cuticle was suggested to contribute importantly in the uptake of chemicals by plants, as the pore structures would offer a rapid access of toxicants to the deeper cuticle parts [18]. However, Li et al. [9] found that the highest intensity of phenanthrene in the adaxial cuticle was nearly twice as much as that in the abaxial cuticle. The impact of this structural difference on the nuclide adsorption is still unknown. In addition, the probabilities for the abaxial and adaxial foliar cuticles to be exposed to contaminants are importantly different, as the probability for the adaxial ones is much higher than the abaxial ones. If so, the referential significance of the results from studies on the mixed cuticles requires further investigation. For this reason, the focus of this study was aimed at the differences in the composition and adsorption behavior between the adaxial and abaxial cuticles. The common dicotyledonous *Photinia fraseri* was selected as the subject leaves, and the composition differences between the abaxial and adaxial foliar cuticles were analyzed through elemental analysis, and Fourier transform infrared spectroscopy (FTIR). Taking the most extensively studied strontium as the representative nuclide [19], a batch adsorption was adopted to explore the adsorption characteristics of the abaxial and adaxial cuticles in an attempt to provide a theoretical basis for the accurate prediction of the nuclide activities in foliar uptake.

## 2. Material and Methods

### 2.1. Samples Collection

**Figure 1:**
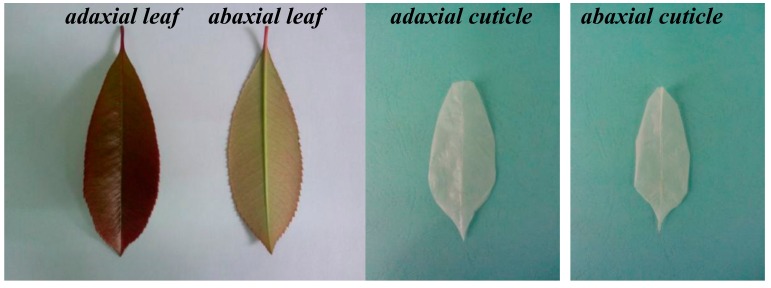
Graph of selected adaxial (PAC) and abaxial (PBC) cuticle as well as adaxial and abaxial *Photinia serrulata* Leaf.

Mature leaves of *Photinia serrulata* (Figure 1) were collected on the campus of Southwest University of Science and Technology, China, and washed with distilled water to remove the dust from the surface. Cuticles were detached using the method described in an earlier report [9]. Then the air dried leaves were weighed and vertically cut in the main vein (in order to facilitate separation), put into the mixture of 30% H_2_O_2_ and glacial acetic acid at 1:1, and heated by a 60 °C water bath until the leaves faded into transparent (about 10 h). The abaxial and adaxial foliar cuticles were carefully peeled off by thin-tipped tweezers while being kept as intact as possible (Figure 1). The separated cuticles were stirred by using a magnetic stirrer to sufficiently remove the margins and mesophyll tissues, rinsed with distilled water again, heated at 60 °C, ground, sieved through a 100-mesh sieve, and kept separately as samples.

### 2.2. The Characterization of Abaxial and Adaxial Cuticles

The leaves of Photinia fraseri were placed on the sample compartment containing conductive paste, and the surface morphology of abaxial and adaxial foliar cuticles were observed via a scanning electron microscope (Leica S440). The CHN elemental analyzer (Vario EL CUBE, Germany) was employed to determine the element contents of C, H, and N in the abaxial and adaxial cuticles, while the oxygen content was calculated by the mass difference. The H/C and (O + N)/C atomic ratios were calculated to evaluate the aliphatic nature and polarity of the selected cuticles. FTIR spectra were recorded in the 4000 cm^−1^–400 cm^−1^ region for a KBr-pellet by a Nicolet FTIR spectrophotometer (Nicolet 5700) with a resolution of 2 cm ^−1^.

### 2.3. Batch Adsorption Experiment

Strontium was chosen as a representative nuclide, and the salt of SrCl_2_·6H_2_O was used to prepare the stock solution of Sr. The adsorption of adaxial and abaxial cuticles was performed with the specific solid (50 mg) to-solution (8 mL) ratios adjusted to achieve 30%–80% removal rate of sorbate at apparent equilibrium in comparison with initial concentration. Each adsorption isotherm consisted of ten concentration points ranging from 1.5 to 100 mg·L^−1^ while each point included the control and calibration. The vials were full-filled with sorbate solution to minimize the headspace volumes of vials, sealed with Teflon screw caps to avoid sorbate’s evaporation, and then agitated in the dark for 1 h (kinetic experiment indicating adsorption obtained equilibrium with 30 min) at 25 ± 0.5 °C in the dark at an agitating rate of 150 r·min^−1^. After agitation, the solution was filtered by a 0.45-µm aqueous phase needle filters and measured by a Perkin-Elmer Analyst 700 (PE700, Waltham, MA, USA) atomic adsorption spectrometer with a wavelength of 460.7 nm. The residual solid (cuticle with and without Sr) was dried for FTIR analysis. Because of the minimal sorption by the vials, and negligible losses from evaporation and degradation (considering the limited equilibrium time), the sorbed amount was then determined by difference in aqueous concentration between nominal aqueous concentration without sorbent and with sorbent.

Surface charge properties of adaxial and abaxial cuticles were measured with zeta potential. Cuticle (2.5 g·L^−1^) was suspended in deionized water or 20 mg·L^−1^ Sr solution and adjusted to pH 1–7, and the zeta potential was obtained by a Zetasizer (Nano ZS90, Malvern, UK). The pH of the cuticle suspension was adjusted using 1 mol·L^−1^ HCl or 1 mol·L^−1^ NaOH. At the same time, the final values of pH, and removal rate of sorbate were measured for the solutions before and after adsorption.

### 2.4. Data Analysis

The amount of Sr adsorbed onto the cuticles ($Q_{e}$) was calculated by mass equilibrium (Equation (1)), and removal rate (*R*) of Sr was calculated according to Equation (2).

(1) $$Q_{e}=\frac{\left(C_{0}{-C}_{e}\right)\;\times \;V}{1000\;\times \;m}$$

(2) $$R\%=\frac{{(C}_{0}{-C}_{e})\;\times \;100}{C_{0}}$$

where $Q_{e}$ is the amount sorbed per unit weight of sorbent (mg·g^−1^), $C_{0}$ and $C_{e}$ are the original and equilibrium Sr concentration in aqueous phase (mg·L^−1^), respectively, *V* is the solution volume (mL), and *m* is the cuticle mass (mg).

Both Langmuir (Equation (3)) and Freundlich (Equation (4)) models were used to describe the sorption isotherm of cuticles.

(3) $$\mathrm{Langmuir}\;\mathrm{model}\;\frac{C_{e}}{Q_{e}}=\frac{1}{K_{L}{\times Q}_{m}}+\frac{C_{e}}{Q_{m}}$$

(4) $$\mathrm{Freundlich}\;\mathrm{model}\;Q_{e}{=K}_{f}{\times C}_{e}^{N}$$

where $Q_{e}$ is the amount sorbed per unit weight of sorbent (mg·g^−1^); $C_{e}$ is the equilibrium concentration (mg·L^−1^); Langmuir $Q_{m}$ (mg·g^−1^) is the maximum sorption amount per unit weight of sorbent, and $K_{L}$ indicates the affinity between adsorbent and adsorbate, as the greater the $K_{L}$, the greater the affinity; $K_{f}$ [(mg/g)/(mg/L)*^N^*] is the Freundlich capacity coefficient, and *N* (dimensionless) describes the isotherm curvature.

## 3. Results and Discussion

### 3.1. The Characterizations of Cuticles

**Figure 2:**
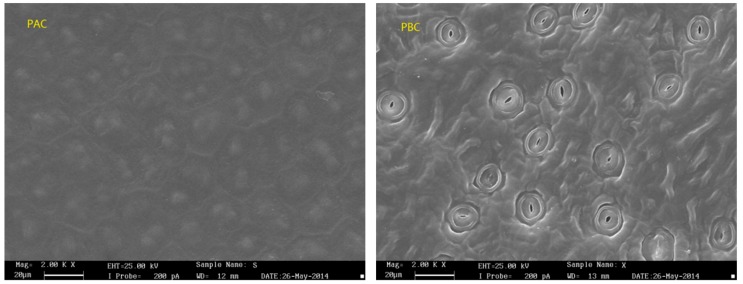
SEM micrographs of adaxial (PAC) and abaxial (PBC) cuticle of *Photinia serrulata* Leaf.

SEM was applied to observe the surface morphology of the abaxial and adaxial cuticles (Figure 2). The adaxial cuticle was slightly wrinkled with bumps of waxy crystal, while protruding stomata was irregularly spread of on the surface of abaxial cuticle. The stomas were approximately 16 μm in diameter and 3.5 μm in opening length with a density of about 70,000/cm^2^, and the stoma area accounted for nearly 10% of the area of the adaxial foliar cuticle.

The yield and chemical properties of the adaxial and abaxial leaf cuticles are shown in Table 1. For one piece of fresh leaf, the amount of adaxial cuticle (7.01%) was a little higher than that of abaxial one (5.78%). The elemental compositions of both cuticles were very similar, with high level of organic carbon (56.88% and 55.60%, respectively). H/C ratio characterized the extent of aromaticity of the samples, while the (O + N)/C ratio characterized the extent of polarity. The H/C atomic ratios were respectively 1.59 and 1.65, suggesting that both the abaxial and adaxial leaf cuticles were highly fatty. The (O + N)/C atomic ratios were respectively 0.47 and 0.50, suggesting high polarities of leaf cuticles [9].

**Figure 3:**
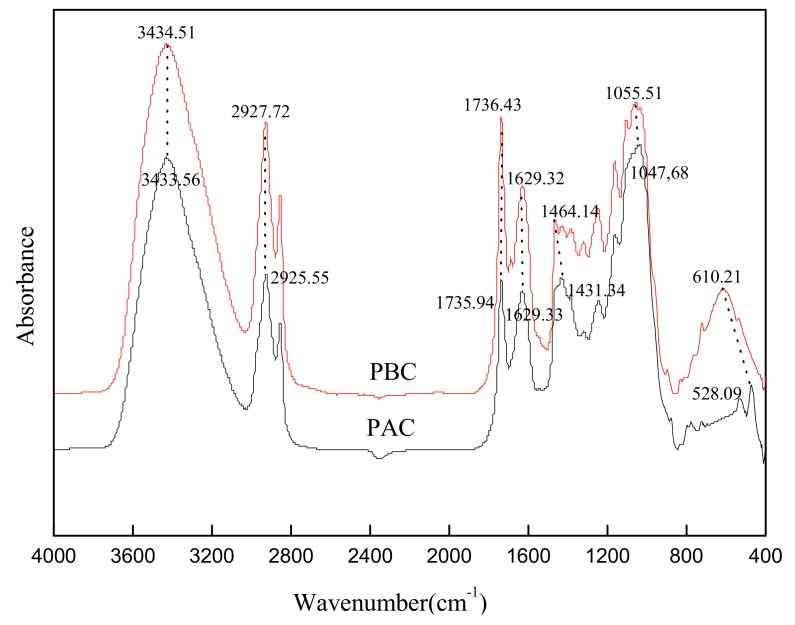
FTIR spectra of adaxial (PAC) and abaxial (PBC) cuticle of *Photinia serrulata* Leaf.

The FTIR spectra of abaxial and adaxial leaf cuticles are shown in Figure 3. The adsorption peaks nearby 2926, 2855, and 1431 cm^−1^ were assigned mainly to CH_2_ units of aliphatic components of the cuticle, including wax, cutin and cutan. The bands at 1736 and 1164 cm^−1^ were the carboxyl C = O and C-O stretching vibration peaks of cutin, respectively. The peaks at 1629 cm^−1^ was assigned to aromatic ring C = C, C = O, mainly derived from polymeric cutan. The bands at 1048 cm^−1^ (1056 cm^−1^) and the surrounding shoulder peaks were assigned to C-O-C and OH, mainly from the polysaccharide components of the cuticle. Generally, the functional group distribution of the adaxial and abaxial cuticles was quite similar in the current study, and distinctively different from the result of Li et al. [9] in that the band intensity of adaxial cuticle was much stronger than abaxial ones (intact cuticle). This was mainly attributed to the different sample shape (cuticle powder in current study while intact cuticle in the related reference).

### 3.2. Adsorption Kinetics

**Figure 4:**
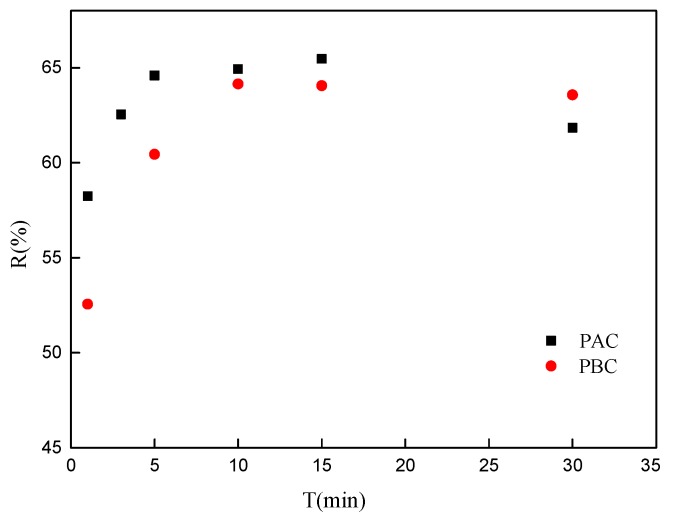
Adsorption kinetic of Sr by adaxial (PAC) and abaxial (PBC) cuticle of *Photinia serrulata* Leaf.

The adsorption kinetics of the adaxial and abaxial foliar cuticles of *Photinia fraseri* for Sr are shown in Figure 4. The adsorption process is a dynamic equilibrium process of adsorption–desorption. The adsorption rate mostly depends on the physicochemical structure of the sample and the nuclide ions characteristics. Adsorption of Sr on the leaf cuticle of *Photinia fraseri* is up to the adsorption equilibrium within 10 min, and the adsorption rate is faster than that of the moss adsorption of Sr [20], but a little lower than almond peel hell [19]. The rapid adsorption of radionuclides in the cuticle has an important effect on the migration and transformation of radionuclides in the environment. On one hand, the rapid adsorption of nuclides onto a cuticle can reduce the environmental mobility of radionuclides, reducing the pollution range of nuclides and decreasing the exposure to nuclides in soil and water environment. In contrast, fast-adsorbed nuclides can be absorbed by plants, and then enter the food chain endangering human health and ecological safety.

### 3.3. Adsorption Isotherm

**Figure 5:**
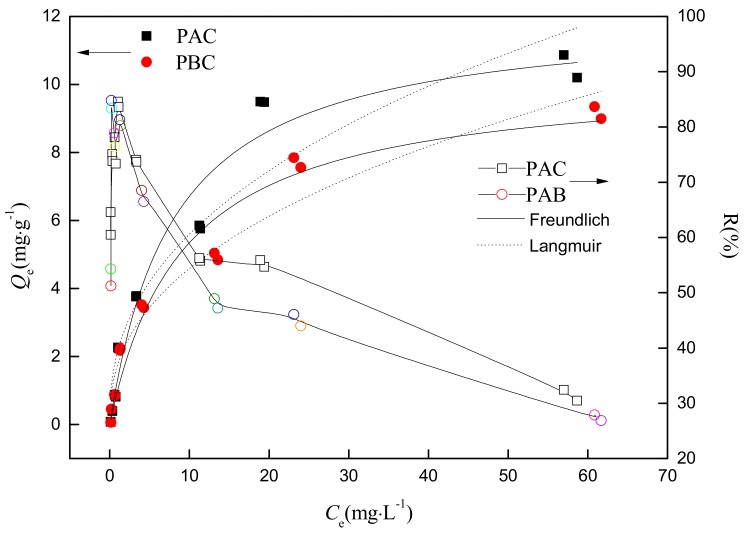
The adsorption isotherm and the adsorption percentage of Sr by adaxial (PAC) and abaxial (PBC) cuticles of *Photinia serrulat* Leaf. The solid and open legends represent the mass of adsorbed Sr ($Q_{e}$) and adsorption percentage of Sr (*R*%) in an equilibrated system.

The adsorption isotherms of the adaxial and abaxial foliar cuticles of *Photinia fraseri* for Sr are shown in Figure 5. The adsorption characteristics of the abaxial and adaxial foliar cuticles were very similar, but represented a significant departure with respect to organic pollutants [9]. With an increase in the initial concentration of Sr, the adsorption amounts onto the abaxial and adaxial foliar cuticles showed an obvious upward trend, while the removal rate firstly increased and then decreased. This is because when the concentrations were relatively low, the collision probability of sorbates and cuticle is small, leading to low removal rate. With increasing of the concentration of Sr in aqueous solution up to ≤1 mg·L^−1^, the accessibility of the cuticles sorbing sites by Sr increased significantly resulting in a growing trend in the adsorption rate. However, considering the limited adsorption sites, there was a gradual downward trend of the adsorption rate with a further increase in the concentration of nuclide ions (≥1 mg·L^−1^).

The regression parameters of Langmuir and Freundlich models of the adsorption isotherm are shown in Table 2. Compared with the Freundlich model (*R*^2^ were 0.92 and 0.96, respectively), the Langmuir model (*R*^2^ were 0.97 and 0.97, respectively) could more accurately describe the adsorption process of *Photinia fraseri* cuticle for Sr. $K_{L}$_(PAC)_ (0.125) and $K_{L}$_(PBC)_ (0.109) were very close, suggesting that the affinity between adaxial and abaxial cuticle and Sr was quite similar. The theoretical maximum adsorption capacity of adaxial cuticle (12.1 mg·g^−1^) was slightly higher than that of abaxial cuticle (10.3 mg·g^−1^), which was similar to the maximum adsorption capacities obtained in experiments (adaxial cuticle, 10.8 mg·g^−1^; abaxial cuticle, 9.3 mg·g^−1^). These results were also close to the sorption capacity of moss (14.0 mg·g^−1^, [20]), root tissues (12.89 mg·g^−1^, [21]) and Sunflower straw (17.48 mg·g^−1^, [22]). The similar adsorption capacity of cuticle and plant tissue in references indicated that both adaxial and abaxial cuticles don’t play a negative role on the absorption of nuclides which was usually considered to be a barrier for the foliar uptake of pollutants.

The specific activity of the radionuclide ^90^Sr which was corresponding to the saturated adsorption capacity was 5.7 × 10 ^13^ Bq·kg^−1^ (adaxial cuticle) and 4.9 × 10 ^13^ Bq·kg^−1^ (abaxial cuticle), classified as high-level radioactive waste. Therefore, the cuticles had a strong retention capacity for the nuclides in the environment, while its high radioactivity is a great safety risk.

### 3.4. Effect of pH on Sr Adsorption

**Figure 6:**
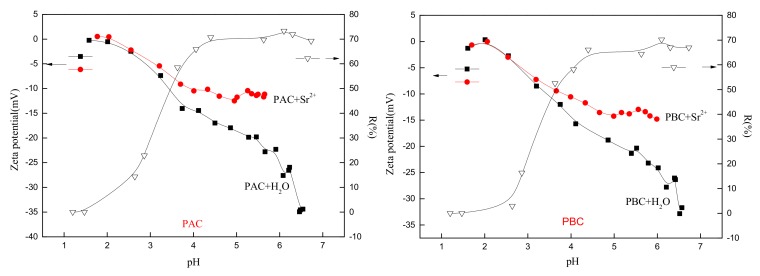
Effect of pH on Sr adsorption and zeta potential of adaxial (PAC) and abaxial (PBC) cuticle with and without Sr loading.

**Figure 7:**
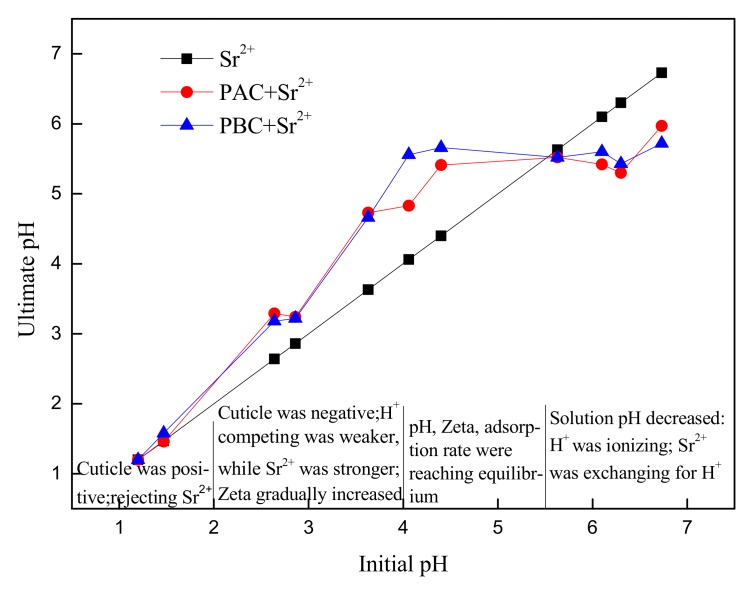
Solution pH before and after Sr adsorption by adaxial (PAC) and abaxial (PBC) cuticle of *Photinia serrulata* Leaf.

The effect of pH on the adsorption behavior of adaxial and abaxial cuticles is shown in Figure 6, and the initial and final pH values of adsorption working solution are also shown in Figure 7. Considering the significance of surface charge properties on metal ion sorption behavior [23], the corresponding zeta potential of cuticle particles with and without Sr loading was also determined (Figure 6). Ionization and protonation of the functional group (such as -O-, -OH, =O, -COOH, -NH_2_) of cuticle particles surface directly changes upon interaction with H^+^ or OH^−^ with the various pH values, resulting different zeta potential [24].

The surface charge characteristics of the abaxial and adaxial foliar cuticles were very similar (Figure 6), and the isoelectric points were around 2.0 before and after the loading of Sr. When the solution pH values were greater than the isoelectric point, the zeta potential of particles changed to a negative value, indicating that the cuticles carried a negative charge. Since Sr carried a positive charge, the two could be bound together by electrostatic attraction. The low isoelectric point of the *Photinia fraseri* cuticle provided a strong adsorption capacity for the cations of nuclides within a large range of pH. The value of the isoelectric point was closely related to the adsorption and accumulation of nuclide ions in the cuticles, and was an important indicator for phytoremediation of nuclide contamination and prevention of agricultural contamination.

When pH value was lower than 2, the removal rate of Sr was nearly zero, and the zeta potential of both cuticles almost remained unchanged after being in contact with Sr [25]. This was attributed to the intensively protonation of functional group (such as -OH, -NH_2_) which made it difficult for Sr and cuticle particle to access each other. The pH value of working solution also remained unchanged suggesting no obvious adsorption of H^+^. With pH value further increasing, the adsorption rate was firstly drastically increased (pH, 2–4) and then gradually came to equilibrium (pH ≥ 4). Corresponding, zeta potential increased slowly after Sr adsorbed. When pH value was between 3 and 4, zeta potential began to drastically increase after Sr loading, and the removal rate also rose rapidly. When pH value was between 4 and 7, zeta potential gradually came to equilibrium after Sr adsorbed and the related adsorption rate reached its equilibrium, suggesting that adsorption behavior of cuticle was importantly affected by the surface charge of sorbents particles. The equilibrium value of zeta potential of adaxial cuticle (−11.5 mV) was greater than the abaxial cuticle (−13.0 mV), indicating a stronger binding with Sr of adaxial cuticle.

**Figure 8:**
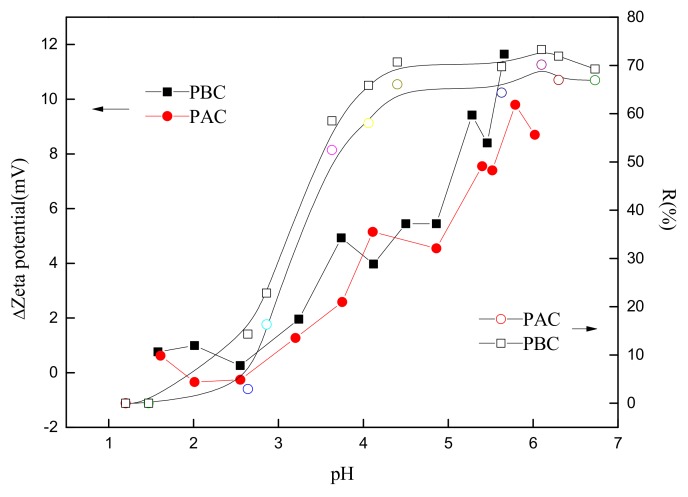
Relationship of removal rate and the change of zeta potential of cuticle before and after Sr adsorption by adaxial (PAC) and abaxial (PBC) cuticle of *Photinia serrulata* Leaf.

The correlation between the adsorption rate and the increase in zeta potential before and after Sr loading (ΔZeta) is shown in Figure 8. The positive correlation between the adsorption rate and increases in zeta potential was exhibited, indicating that the key mechanism of adsorption was electrostatic attraction and complex reaction rather ion exchange. The increase in zeta potential represented the growth of the surface charges of the adsorbent, and the ion exchange was an equal value exchange that would not change the amount of surface charges. Hence, the reason why the amount of (negative) charges was reduced after the adsorption was that part of the negative surface charges were counteracted by the electrostatic attraction and complex reaction with the cations.

The pH values of the abaxial and adaxial cuticles of *Photinia fraseri* before and after Sr were adsorbed are shown in Figure 7. When the pH value was less than 5.5, the pH of the solution increased after Sr were adsorbed, indicating that the negatively charged cuticles had adsorbed the H^+^ in the solution. When pH value was greater than 5.5, the pH of solution dropped. The decline in pH was not caused by the hydrolysis of Sr, because the hydrolysis constant of Sr logβ_1_ = −13. 29 and logβ_2_ = −28.51, suggesting that when pH was between 1 and 7, the Sr(OH) ^+^ and H^+^ generated from hydrolysis could be ignored [26]. There were two aspects of the pH reducing effect: on the one hand, H^+^ were separated by the -OH and -COOH acidic functional groups; on the other hand, the ion exchange occurred between Sr and H^+^, substituting H^+^. When the pH value was greater than 5.5 (H^+^ ≤ 3, 16 × 10 ^−6^ mol·L^−1^), the pH began to fall, indicating that the ion exchange was very weak, far less so than the physical electrostatic interaction.

### 3.5. FTIR Spectra of the Cuticles before and after Loading with Sr

**Figure 9:**
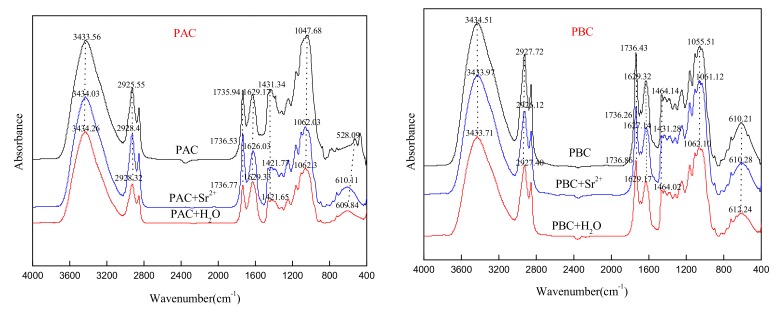
FTIR spectrometer of cuticle before and after Sr adsorption by adaxial (PAC) and abaxial (PBC) cuticle of *Photinia serrulata* Leaf.

The infrared spectrum of the abaxial and adaxial foliar cuticles of *Photinia fraseri* is shown in Figure 9. The characteristic functional groups in the abaxial and adaxial foliar cuticles did not change importantly before and after the adsorption for Sr, suggesting that there was no chemical precipitation in the adsorption of cuticles for Sr. For PAC, after Sr were adsorbed, the peaks at 2926, 2856, 1736, 1464, 1421, 1246, 1164, and 1062 cm^−1^ were intensified, showing that C-H, -COOH, and C-O-C were all involved in the adsorption; while the peak at 1629 cm^−1^ moved towards the low wave-number to 1626 cm^−1^, following Sr adsorption, the H^+^ in carboxyl groups were replaced, causing a decline in the vibration intensity of the C=O bond and a reduced wave number at the maximum adsorption peak. For PBC, the peak intensities almost remained unchanged, but the one at 1464 cm^−1^ moved towards the low wave-number by 4 cm^−1^, while those at 2927, 1629, and 1062 cm^−1^ moved towards the low wave-number by 1–2 cm^−1^, showing that C-H, phenolic hydroxyl group, and C-O-C might have undergone ligand complexation and ion exchange with Sr.

## 4. Conclusions

1. The elemental composition and FTIR spectra for both adaxial and abaxial cuticles were quite similar and both show high affinity (H/C, 1.59 and 1.65) and polarity ((O + N)/C, 0.470 and 0.499). Adsorption of Sr onto adaxial and abaxial cuticles was up to equilibrium with 10 min, and both adsorption isotherms fit well with Langmuir model (*R*^2^, 0.97 and 0.97).
2. Neither adaxial nor abaxial cuticles play a negative role on the absorption of nuclide which was usually considered to be a barrier for the foliar uptake of pollutants, but showed a strong retention capacity for the nuclides in the environment. The maximum adsorption capacity of PAC was 12.1 mg·g^−^^1^, little higher than that of PBC (10.3 mg·g^−^^1^).
3. The adsorption of Sr increased with the increase of pH, and the maximum was attained when pH ≥ 4. Electrostatic attraction was demonstrated to be the main mechanism of Sr adsorption onto PAC and PBC, and the similar adsorption of adaxial and abaxial cuticles was consistent with their similar isoelectric point (≈ 2).
4. Importantly, the characteristic functional groups in the abaxial and adaxial foliar cuticles did not change before and after the adsorption for Sr, suggesting that there was no chemical precipitation in the adsorption of cuticles for Sr.

## Figures and Tables

**Table 1:** Yields and elemental analysis of adaxial (PAC) and abaxial (PBC) cuticle of *Photinia serrulata* Leaf.

Sample	Yield/%*^a^*	C/%	H/%	N/%	O/%*^b^*	H/C	(O + N)/C
adaxial cuticle	7.01	56.88	7.55	0.45	35.12	1.59	0.47
abaxial cuticle	5.78	55.60	7.63	0.45	36.32	1.65	0.50

Notes: a Yield of cuticles was calculated to the percentage contents of correspondingly fresh weight of leaves. b Oxygen content was calculated by the mass difference.

**Table 2:** Regression parameters of Langmuir and Freundlich model for the sorption of Strontium by adaxial (PAC) and abaxial (PBC) cuticle of *Photinia serrulata* Leaf.

Sample	Langmuir Regression Parameters			Freundlich Regression Parameters		
	*K*_L_/(L·mg^−1^)	*Q*_max_/(mg·g^−1^)	*R* ^2^	*K*_f_/(mg/g)/(mg/L)^N^	*N*	*R* ^2^
PAC PBC	0.125 ± 0.023	12.1 ± 0.71	0.97	2.15 ± 0.32	0.415 ± 0.044	0.92
	0.109 ± 0.021	10.3 ± 0.64	0.97	1.76 ± 0.20	0.417 ± 0.032	0.96

Notes: $Q_{e}$ is the amount sorbed per unit weight of sorbent, mg·g^−^^1^; $C_{e}$ is the equilibrium concentration, mg·L^−^^1^; $K_{f}$ [(mg/g)/(mg/L)^N^] is the Freundlich capacity coefficient; and *N* (dimensionless) describes the isotherm curvature. *R*^2^ is regression coefficient.
